# Influences on seeking a contraceptive method among adolescent women in three cities in Nigeria

**DOI:** 10.1186/s12978-020-01019-1

**Published:** 2020-10-28

**Authors:** Elynn Kann Sanchez, Ilene S. Speizer, Elizabeth Tolley, Lisa M. Calhoun, Clare Barrington, Adesola O. Olumide

**Affiliations:** 1 Department of Maternal and Child Health, Gillings School of Global Public Health, UNC, Chapel Hill, North Carolina USA; 2 Carolina Population Center, University of North Carolina-Chapel Hill, Chapel Hill, North Carolina 27516 USA; 3 FHI 360, Durham, North Carolina USA; 4 Department of Health Behavior, Gillings School of Global Public Health, UNC, Chapel Hill, North Carolina USA; 5 Institute of Child Health, University of Ibadan, College of Medicine, Ibadan, Nigeria

**Keywords:** Adolescents, Sub-Saharan Africa, Contraception

## Abstract

**Background:**

Despite international support for increasing access to contraceptives among adolescents, gaps in use still exist worldwide. Past research has identified barriers to use across all levels of the socioecological model including restrictive policies, a lack of youth friendly services, and knowledge gaps. This study was conducted to further identify influences on contraceptive use among adolescent girls in Nigeria in hopes of guiding future policies and programs.

**Methods:**

In 2018, 12 focus group discussions (FGD) were conducted in three cities in Nigeria with young women ages 15–24 with the objective of determining what and who influence adolescents’ contraceptive seeking behaviors. A vignette structure was used to identify perceptions on injunctive and descriptive community norms that influence adolescent contraceptive behaviors. The FGDs were conducted by members of the University of Ibadan Centre for Population and Reproductive Health (CPRH) and analyzed by a researcher at the University of North Carolina-Chapel Hill’s Carolina Population Center using a thematic analysis approach.

**Results:**

Participants identified community level resistance to sex and contraceptive use among unmarried adolescents though also acknowledged that these adolescent behaviors are still occurring despite established norms. Concerns about side effects and the preservation of fertility were frequently attached to contraceptive use and pointed to as a reason for community resistance to contraceptive use among this population. Participants saw peers, parents and partners as influencers on a girl’s decision to seek a method, though each were believed to play a different role in that decision.

**Conclusion:**

The findings show that that despite barriers created by established injunctive norms, young women with a supportive social network can access contraceptive methods despite these barriers. By harnessing the influence of peers, partners and parents, the Nigerian family planning efforts can strive to improve the health and well-being of young people.

## Plain English summary

Using focus group discussions (FGD) with young women ages 15–24, this qualitative study sought to understand the perceived influences on contraceptive use among adolescent girls in Nigeria. Building on past research, this study identifies known barriers across the socioecological model, particularly a resistance to sex and contraceptive use among unmarried adolescents. When considering to use a contraceptive method, concerns around side effects and preserving fertility were discussed as reasons many choose not to use a method. This was particularly true for young women who had not yet had children. The participants also discussed the role of peers, partners and parents on the girl’s decision, and how this influence is dependent on the closeness of the relationship. If supportive, these relationships were seen as an aid to contraceptive use. Unsupportive relationships were not spoken of as dissuasive to use. Instead, girls with an unsupportive network were likely to navigate accessing a method alone, leaving her vulnerable to the known barriers for adolescent girls.

## Introduction

A pregnancy is a life altering event, particularly among adolescents as it is a significant reason why young girls globally drop out of school and fall into poverty [1, 2]. Additionally, adolescent pregnancy comes with an increased risk of complications and preterm birth resulting in maternal morbidity and mortality, and remains the leading cause of death among adolescents [3]. It is estimated worldwide that half of all adolescent occurring pregnancies are unintended [1]. Strategies to reduce early pregnancy promote increased access to contraceptives [4–7]. Despite the recognition of adolescent contraceptive needs, there are still large gaps in contraceptive use within adolescent populations.

An important indicator of this gap is the unmet need for family planning, which is usually measured as the percent of fecund, sexually active women who want to limit or delay childbearing (beyond 2 years), but are not using a contraceptive method [8]. In 2015, rates of unmet need among married women ages 15–24 averaged as high as 29.3% in some regions, while rates among unmarried women of the same age averaged around 40% [8]. Among the 61 countries analyzed in the 2015 DHS Comparative Report, it is estimated that 33 million adolescents ages 15–24 have an unmet need for contraception [8]. Achieving universal contraceptive method coverage for sexually-active adolescents who desire to use a method would lower the number of unplanned pregnancies by an estimated 63% per year, greatly lowering the burden of negative outcomes among this population [3].

In understanding the unmet need gap, researchers aim to identify barriers that prevent adolescents and young women from seeking a contraceptive method across all levels of the socioecological model (SEM). The SEM is used to guide the creation of public health programs and policies to ensure they address the interacting and layered social and environmental factors influencing human behavior. The model, originally created by Urie Bronfenbrenner in the 1970s, is widely used by international family planning stakeholders [9]. At the policy level, laws against contraceptive access for adolescents exist around the world. For example, in 2017 the Population Reference Bureau (PRB) analyzed the legal frameworks of 16 countries for laws that restricted contraceptive access based on age and parental or provider authorization. In Nigeria, the report found that no law currently protects against providers requiring authorization or parental consent. Additionally, though laws exist that support access regardless of age, there are no provisions that support the coverage of a full range of methods no matter the age [10]. This is just one example, as despite international efforts to promote more supportive legal environments at national and state levels, supportive laws for adolescent and youth access to contraception seem to be lacking [10]. At the community and organizational levels, religious restrictions, provider bias, community stigma, and lack of youth friendly services have been found to negatively impact adolescents’ decisions to seek a method [11–15]. Interpersonal factors, such as a perceived lack of support from a partner, can also limit the social support that one may need to seek a method [3]. Finally, at the individual level, a study by Guttmacher demonstrates that among young women ages 15–24, infrequent sex, potential side effects of methods, and delayed return of menstruation after birth are the common reasons for non-use of a method [3]. In addition, informational barriers, whether at the interpersonal or individual levels, have been shown to challenge adolescents’ understanding of what resources are available to them [10]. This paper will focus mainly on interpersonal and individual level factors, though the understanding of barriers across the SEM assists in highlighting the context that makes combatting the breadth of challenges complex.

We use qualitative data determine what and who influences adolescent girls’ contraceptive seeking behaviors. By answering these questions, this paper strives to identify influences that can be targeted by future programs and policies in Nigeria to improve adolescent and youth access to family planning methods and services.

## Context

**Fig. 1:**
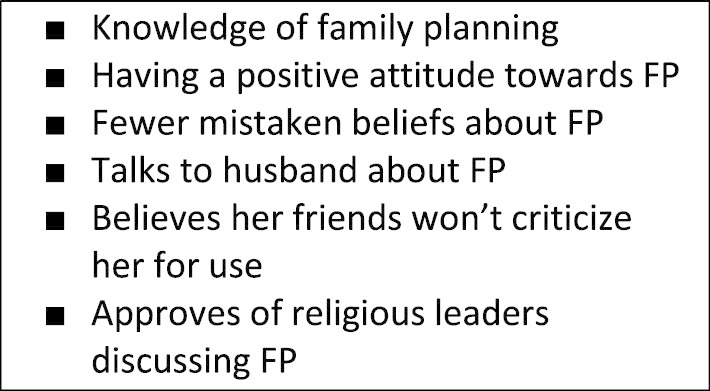
NURHI ideational factors

Focus group discussions (FGDs) were conducted as part of a larger study which investigated the sustainability and impact of the Nigerian Urban Reproductive Health Initiative (NURHI) Phase one activities which ran from 2009 through early 2015. The program was led by the Center for Communication Programs at Johns Hopkins University and funded by the Bill & Melinda Gates Foundation. NURHI was designed to target ideational factors (see Fig. 1) that could increase the demand for and use of modern contraception among women in six urban sites of Nigeria [15]. NURHI also strengthened service delivery and undertook advocacy programs that sought to increase access to and improve quality of contraceptive services in an attempt to support the increased demand for those services, as the ideational factors shifted [16]. The NURHI program was implemented in Ilorin, Kaduna, Ibadan, Benin City, Zaria and Abuja. This qualitative study was conducted in two of the six intervention cities (Ilorin and Kaduna), as well as a comparison city, Jos, in Plateau state.

When discussing adolescent reproductive health, Nigeria is a strong case study, as more than half of the population is under the age of 24 years [17]. As of 2017, 55% of Nigerian youth live in urban areas; a population that faces different challenges than their rural counterparts [17]. Nigerian data suggest that urban adolescents and youth are initiating sexual activity before marriage with the average age at first marriage being 23.7 years, while the average age of first sex is 19.2 years [17]. Additionally, 2017 PMA2020—Round 4 data show that the average age of first contraceptive use among urban adolescents throughout Nigeria is 25 years, leaving an almost 6 year gap between first sexual initiation and contraceptive use [17]. The same round of PMA2020 data found that among adolescents and youth (ages 15–24 years) the use of modern contraceptive methods (e.g., condoms, oral pills, injectable, IUD, etc.) is 7.4% among married women, while it is 36.0% among unmarried, sexually active women; 56.6% of which is accounted for by male condoms [17]. In 2013, the Nigerian national total fertility rate was 5.8 children per women ages 15–49; a slight decrease from 6.1 children in 2010 though still high in comparison to other countries in the region [18, 19]. Studies continue to find a pronatalist culture in Nigeria, particularly within married populations which could be contributing to this discrepancy in contraceptive use between married and unmarried youth [19, 20].

## Methods

In June and July of 2018, 18 FGDs were conducted as a part of the NURHI Sustainability Study. Participants were recruited through a convenience sample by community contacts in each city; participants were grouped by age (15–24 and 25–39 years), religion (Christian and Muslim) and marital status (currently married and unmarried). Because of our focus on understanding factors influencing contraceptive behaviors among young people, this analysis focused on the 12 FGDs with participants ages 15 to 24 years, which included four FGDs in each city (Kaduna, Ilorin, and Jos). There were eight to 13 participants in each FGD, leading to a total of 117 participants across the 12 FGDs (Table 1). As shown in Table 1, the FGD were undertaken with homogenous groups such that married young people by religious affiliation were in a group as were unmarried young people; this was determined to be an appropriate grouping to support open discussion.

**Table 1:** Focus group discussion demographics

FDG number	Location	Marital status	Religion	Number of participants	Average age of the participants (by city)
1	Ilorin	Married	Christian	12	20.4 years
2	Ilorin	Married	Christian	9	
3	Ilorin	Unmarried	Muslim	9	
4	Ilorin	Married	Christian	13	
5	Jos	Unmarried	Christian	10	20.9 years
6	Jos	Unmarried	Muslim	10	
7	Jos	Married	Muslim	10	
8	Jos	Married	Christian	10	
9	Kaduna	Married	Christian	8	20.9 years
10	Kaduna	Unmarried	Christian	9	
11	Kaduna	Unmarried	Muslim	8	
12	Kaduna	Married	Muslim	9	

The discussion guide used a vignette structure, which presented a fictitious young woman in their communities trying to access a contraceptive method. Vignettes have been used in previous research to assess beliefs and social norms on potentially challenging topics, enabling participants to feel comfortable sharing their perspectives through the life of the individual in the vignette [21, 22]. The moderator delivered the following vignette at the start of the discussion,

*“A 16-year-old girl from your community is dating her boyfriend who is 17 years old. They decide to start having sex together. Then the girl learns that her friend is pregnant. She learns about modern contraception, such as condoms, daily pill, injectables, and implants, and is thinking about starting to use modern contraception since she doesn’t want to get pregnant like her friend.”*

After reading the vignette to the participants, the facilitator asked them to respond to a series of questions on their attitudes toward the scenario. The FGDs were held in English, Hausa and Yoruba, depending on participant and group preference.

A team of 19 members of the University of Ibadan's Centre for Population and Reproductive Health (CPRH) collaborated to lead and assist the FGDs. Prior to data collection, the CPRH team participated in trainings focused on best practices in qualitative research, including how to manage the participants and encourage participation from all. Once completed, the FGDs were translated, transcribed and the de-identified transcripts were shared with the research team at the University of North Carolina Chapel Hill’s Carolina Population Center. To begin the thematic analysis process, the transcripts were reviewed to identify themes within the text using handwritten notations in the margins of the transcripts. A codebook was created using these notations and codes were developed specifically related to the research questions. The transcripts were uploaded to Atlas.ti where each transcript was coded. After the first round of coding, Excel matrices were utilized to observe the spread of the assigned codes to further identify patterns in main themes, including variations between demographic groups and locations. A second round of coding was conducted to document newly identified themes, and the Excel matrices were updated to reflect the new patterns.

The discussion guides were structured to examine the participants’ perceptions of the established injunctive and descriptive norms that exist within the community, with injunctive norms representing behaviors that are believed to be approved by the community and descriptive norms being behaviors that are believed to actually be happening around them [23]. Throughout the coding process, codes for injunctive and descriptive norms were applied to text segments to identify what participants thought the character should do (injunctive), and what they believed she would do (descriptive), a distinction that arose widely throughout the transcripts.

## Results

**Fig. 2:**
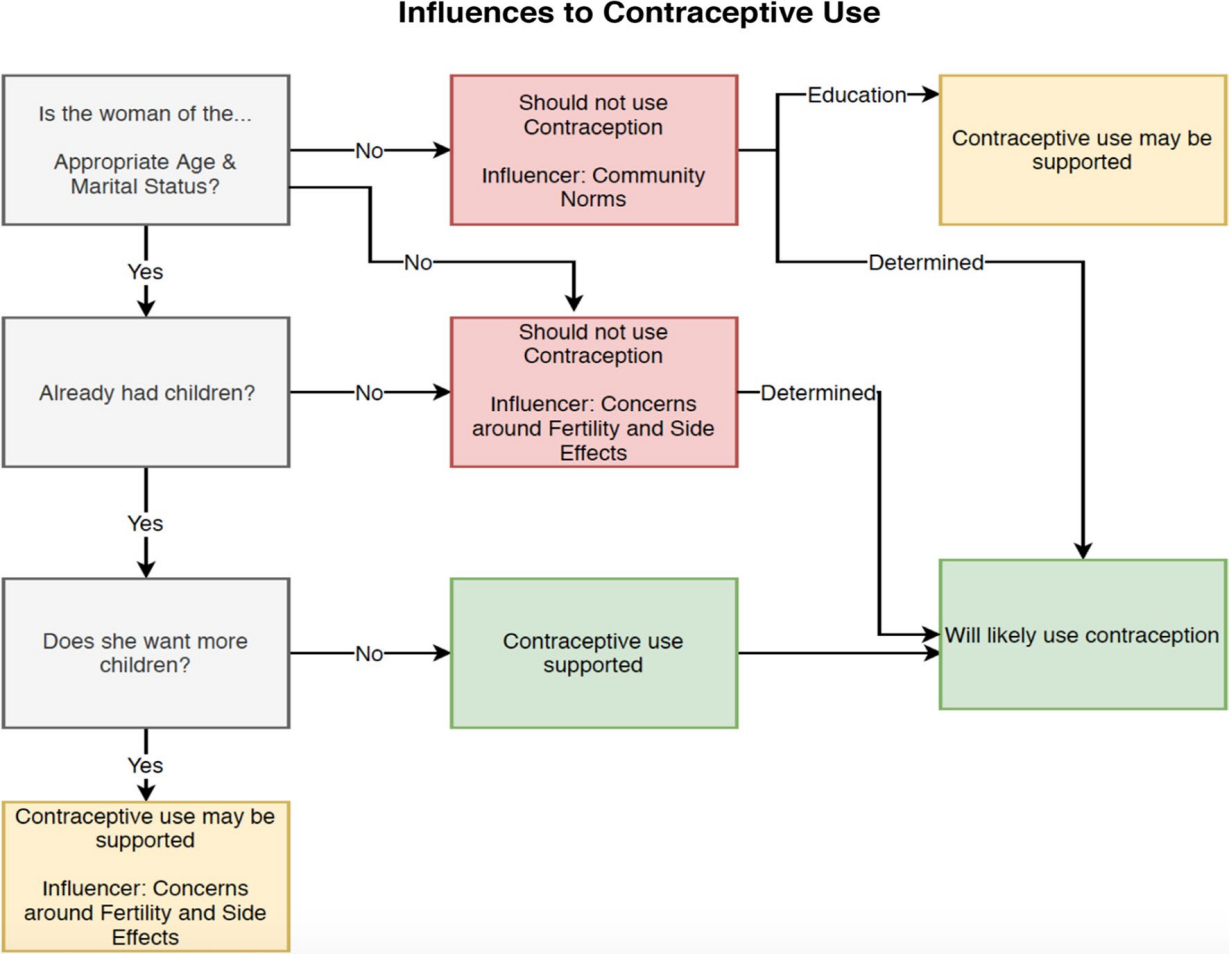
Conceptual model

The results are organized based on the conceptual model found in Fig. 2, which is a synthesis of the analysis. The FGDs all began with discussions around the inherent relationships between sex and contraceptive use, and how support for both behaviors was restricted by the character’s age and marital status. Participants expressed concern around the potential side effects of using contraceptives and believed that the preservation of fertility should be a priority. These perspectives created a picture of what the participants believed the character *should do* based on the approved social context. However, participants also acknowledged that these actions would not always align with what the character *would do,* with education being presented as one potential motivator for this deviation from the established social norm. Finally, the participants described those identified as influencers in the character’s decision to seek care, including peers, parents, and partners.

### Appropriate context—age, marriage and sex

Participants grounded their discussion of the character’s contraceptive use initially by expressing resistance to the character’s choice to have sex due to her age and marital status. Participants in each of the FGDs began with statements in opposition to the character’s sexual activity, frequently describing the character as *wayward* or a *prostitute* due to this behavior. This discussion of sex was initially separated from the character’s desired contraceptive use, as the participants quickly established that due to cultural and religious norms her decision to have sex was not supported. Resistance most commonly related to the girl’s age, with many sharing the sentiment of an unmarried, Christian woman from Kaduna who said, “*She is 16, I think she is not up to that age*.” For participants, there was clear resistance to sexual behavior at 16 specifically, but there was very little discussion about the specific age at which these behaviors would be approved. One statement that alluded to this idea was made by an unmarried, Christian woman from Ilorin who said,

*“The community view will be like, what is she looking for because her community here feels under-19, under-18, are still kids so what is she looking for. The advice they will give her will be to prevent sex or to even stop dating.”*

Here, we see the participant grappling with the idea of the age at which this character would be considered an adult or able to have sex and make her own decisions. Whether a legal or perceived definition of adulthood, this quote relays that there is a norm established around the age of adulthood.

The resistance to sex was also compounded by the character’s marital status, as participants frequently put this forward along with age as a reason against her choice to have sex, as one married, Christian woman from Kaduna stated below,

*“Now she can marry sixteen, seventeen; they can marry now, in this generation now she can marry. Let her just make up her mind now to be married because if anything happens now, she will be married.”*

In this quote, we see the woman suggesting that if the character were to be married, then the decision to have sex would be acceptable, even at the otherwise unacceptable age of 16.

While the participants established that the character was not at the “right” age or marital status to have sex, they similarly attached her contraceptive use to her sexual activity, thus requiring the same prerequisites. The idea of contraceptive use was inherently attached to sex so the two ideas were frequently combined, as exemplified by an unmarried, Christian woman from Ilorin who said,

*“I'm not sure they are going to accept it [contraception] because someone at the age of sixteen is not even meant to be having sex in Nigeria. That's all we believe.”*

Discussions of age as a criterion for contraceptive use occurred in all of the FGDs, aligning with the above belief that this is a norm across Nigeria. Similarly, participants identified marriage as a criterion for support of contraceptive use, as an unmarried, Christian woman in Jos stated,

*“…others will feel ah this girl is still a teenager, why will she start looking for family planning and all that when she is not married.”*

Statements establishing the appropriate social context in which the character’s contraceptive use was supported by the community were identified across all the FGDs. Based on the results of the coding process, Muslim participants were twice as likely as Christians to raise marital status as a concern for both sex and contraceptive use. The lack of acceptance of sexual behavior outside the context of marriage within the Muslim population was discussed widely by participants from both religions, with one married Muslim woman from Jos sharing,

*“Even if she meets her friends, it’s not like they know the side effects nor do they know if there are varieties [of methods] in order to give her advice. So, it is expected she tells her parents the truth…It is better they get her married. That is the better [way] that will protect the community from being totally ruined.”*

Generally, the FGDs with the Muslim participants made it clear that they would prefer that the character get married instead of using a contraceptive method.

Despite these established norms, there was also an acknowledgement by all participants that adolescents in the community are having sex and accessing contraceptives, a sentiment which is best encapsulated by an unmarried, Christian woman from Ilorin who said,

*“I heard that family planning prevents pregnancy and it's used for women, married women, who want to stop giving birth. But girls of nowadays are already doing it.”*

Being young themselves, participants frequently discussed what the character should do within the established social norms, while also acknowledging that what she would do would not always conform to these guidelines. Participants referred to the character as being ‘determined’ and shared sentiments like the one said by a married, Muslim woman from Kaduna,

*“Even if they talk, she has already made up her mind…So what anybody can say or would not say, she will do what is in her mind because she has already set her mind.”*

Though mentioned across the FDGs, unmarried participants more frequently acknowledged this separation between the character’s likely actions compared to established norms; most mentions of this ‘determined’ mindset were made by unmarried participants.

### Future fertility and contraceptive side effects

While established cultural norms influenced the perceived appropriate context for a girl to use a contraceptive method, side effects and the preservation of fertility served as tangible concerns that support the reasoning behind these norms. Concerns around side effects were discussed universally across all FGD in all regions, with slightly more mentions among unmarried participants. Comments commonly mirrored ones like this sentiment expressed by an unmarried, Muslim woman in Ilorin who said,

*“It is not good for a 16 year old to use modern contraceptives because it could have side effects when she’s grown up.”*

Like this woman, other participants commonly discussed fertility concerns in connection with social norms, almost as a justification for why the norms exist. Participants discussed the threat that infertility could place on a future marriage, a concern that made using contraceptives not worth the risk. One married, Christian woman from Kaduna touched on the intersection of age, parity and fertility protection when she said,

*“To me, the age of sixteen is too early for her to take family planning because at times that family planning, I think it has its own side effect, (it) is when you finished giving birth to your children that’s when you have family planning.”*

Like this woman, participants indicated that contraceptives should not be used until a woman reaches her desired parity to avoid the potential physical effects of using a method. Participants talked about side effects through sharing stories of friends or family who had suffered short- or long-term side effects from using a method. Side effects unrelated to fertility like weight gain were mentioned, though most of the discussion centered on fertility concerns. While these concerns were presented as a reason the character should not use contraceptives, participants still acknowledged that this information may not fully impact her decision. For example, while some women might consider side effect information as a deterrent to contraceptive use, those accessing a contraceptive would use this information to select a safer method and avoid potentially riskier methods while still preventing pregnancy.

### Protecting education

The character’s educational pursuits were discussed as a motivator for contraceptive use among adolescents, as well as a reason to support contraceptive use among those previously opposed. Discussions of education were particularly common among participants in Ilorin, who mentioned it as a supportive factor twice as often as participants in Kaduna or Jos. Regardless of the city, education was an alternative motivator for contraceptive use besides early sexual behavior. An example of this idea occurred when one unmarried, Muslim participant from Ilorin acknowledged that opinions on the girl’s behavior would not prevent her from using a method, the moderator probed on why she thought this, to which she replied:

*“Participant: Since she will not continue with it forever because she would still want to give birth.*

*Moderator: Oh, you think it is just for a short while so as to prevent pregnancy.*

*Participant: Yes. She may want to further her education or learn a trade.”*

In this example, the participant viewed contraceptive use as temporary so the character could complete her education, while understanding that she may still want children in the future. Education was understood by some participants as something that should be protected and pursued and was given as a reason why the participants should avoid sex. However, with acknowledgment that sex was occurring, education was also one of the few factors that could negate pressures around age or marital status to generate support for accessing a method among community members.

### Influencers

Throughout the contraceptive decision-making process, the girl’s parents and peers were identified as the most likely people to influence her decision. Mentions of her partner were much less common, and the role of a provider was mostly discussed in connection with the decision around her choice of method, not the decision to use contraception.

#### Peer influence

Participants described peers as being people girls looked to for modeled behavior around sex and contraceptives, as well as a source for contraceptive information. Though discussed throughout, the most frequent mention of peer influence occurred in Ilorin, and Christian participants across all FGDs were over twice as likely to discuss the influence of peers compared to their Muslim counterparts. Unlike some of the other influencers, participants believed that the character *would* turn to her peers, and that those who the character surrounded herself with would dictate how she moved forward. Participants pointed to the character’s friend’s pregnancy as evidence that the character would see sex as normal. For some participants, this was a reason to recommend keeping girls away from certain peers, to prevent exposure from sexual behavior before she is considered old enough, especially outside of marriage. For example, one married, Christian woman from Kaduna introduced this idea by stating,

*“She is sixteen, she won’t see her friend doing something, let her avoid it. The more she sees her friends, they are flirting, doing some things and all that, she too will like to put herself there.”*

Participants across FGDs acknowledged that the girl’s actions could be influenced by what she believed her friends were doing, though this can depend on who her friends are. This applied to both sex and seeking contraceptives, as an unmarried, Christian woman from Jos said,

*“So, if she has positive thinkers as friends they will be able to at least change her mind against doing it [using contraceptives]. And if her friends are just the way she is, they can even strengthen her more, and even expose her to more contraceptives that she can use.”*

The influence described here was a perspective shared by many participants, though this participant also suggested that peers could change her mind against contraceptive use if they did not agree with her actions. This type of verbal influence or resistance was not widely discussed, with the majority of mentions of peer influence referenced as a mirroring of behavior instead. Where participants did see more of this verbal influence was in the sharing of stories between peers about contraceptive methods, particularly about which methods had produced side effects.

#### Parental influence

Parental influence on a girl’s contraceptive use was complex, as parents were seen as the expected enforcer of social norms around sex and contraceptive use. Participants placed the responsibility on parents to start conversations to establish what the character *should do,* and several participants even placed blame on the character’s parents for her decision to have sex and seek a contraceptive. For example, in referencing what the community would think of the character’s actions, one unmarried, Muslim woman from Ilorin said,

*“It could be traceable to a family member or parents. Community members would probably think she is following the footsteps of her parents…most likely her mum.”*

The pressure put on parents disincentivizes them from supporting a daughter’s access to a safe method. The participants explained that this pressure would make a girl’s decision to approach her parents for advice challenging. The influence of parents was more frequently discussed as something the character *should* seek, rather than what she *would* seek, suggesting that community pressures prevent adolescents from actually turning to this relationship.

Despite the social pressure that challenges this relationship, participants did identify parents as a source where girls could access accurate information. Most commonly, parents were perceived as the ones to explain the disadvantages of contraceptives and early sex. Though a few participants also suggested that parents can be a source of method-related information. As a married, Christian woman from Jos said:

*“Is (it) better for us to tell the child the truth? Let’s look at it this way—if we should tell her the truth, abstinence is not (for) all of us, or not everybody can do it. To some extent, we have, they have friends definitely, so if you as a parent will tell her not to do this if she goes out it will be another thing. So we should just advise them in a way that they will understand, and (it) is good to some extent is good for her to use the contraceptive.”*

This woman reflects a dilemma mentioned by others that parents should be a resource in order to ensure that girls are getting accurate information. Yet, we still see hesitance in fostering that communication line. Participants grappled with the need to expose the character to information about sex and contraceptives to promote safe practices, while also connecting this exposure through peers’ to mirrored action that may not be safe.

#### Partner influence

Though discussed the least frequently, participants described partners as an important source of influence, particularly when choosing a method. While the character decided whether to follow her peers or turn to her parents, participants described the partner as a critical player in the decision to use a method and which type of method to use. This is clear in one quote from an unmarried, Christian woman from Jos who said,

*“For me, the person that I think will influence her is her boyfriend, because definitely before she starts thinking of that she talked to him about it, so she will go and do [access contraception], leave everything unto him to choose.”*

When discussing the influence of partners, participants commonly described partners in a decision-making role, as indicated above. Further, a few participants also relayed a high level of mistrust in the character’s partner and thus may not expect the partner to be involved in the decision-making. Several participants recommended that the character avoid sex because her partner would leave her if she became pregnant, again, illustrating some level of distrust of partners. Alternatively, participants also suggested that partners could help remove barriers for the character, particularly by covering the cost of a method. When, partners were discussed, it was most frequently by married participants, though there were no regional or religious differences. Overall, the partner was described as an important influence throughout the contraceptive seeking process, and someone who would play a large, positive or negative, role in the decisions that were made along the way.

## Discussion

This study sought to determine what and who influences adolescent girls’ contraceptive seeking behaviors. We found that despite community level disapproval, young women perceive that unmarried adolescents, including those aged 16, like the character in the vignette, are having sex and accessing contraceptives, and turning to peers, parents and partners for physical and emotional support for their actions or guidance on the best steps to meet sexual and reproductive health needs. We found that adolescents are aware of injunctive norms in their community that dictate what they should do, and that the FGDs participants linked community-level disapproval towards contraceptive use to a lack of acceptance of unmarried and young sexual behavior. When considering the motivators behind this disapproval, marital status was discussed twice as often among unmarried participant FGDs compared to FGDs with married participants (as determined by the distribution of codes between the groups), potentially indicating a greater perceived concern by those who may be facing this barrier themselves. Participants also shared concerns about the safety of contraceptive methods and the potential influence of contraception on fecundability. For young women already using a method, this concern appears to influence the type of method they choose, while for new users it may guide whether or not they decide to use a method. Furthermore, educational pursuits and descriptive norms constructed by friends serve as motivators that promote adolescents looking to supportive influencers to guide the process of accessing a method.

The identified influence of peers and parents reinforces the idea of the “popular health sector,” introduced by anthropologist Arthur Kleinman in 1980 [24]. Along with the modern medical (professional) and the folk medical sectors, the popular sector are the social networks and cultural influences sought for health care guidance. The influencers that comprise this group including peer, parent and community members have been widely researched for their impact on young women’s health decisions [25–27]. This idea came out strongly in the results, with decisions to seek contraceptive care being grounded in the influence of the people and cultural influences surrounding the girl. In discussions about different contraceptive methods, FGD participants themselves frequently shared stories they had heard about or experienced with certain methods, exhibiting the same type of reciprocal relationships they described between the character and her peers. Participants’ engrained perspectives on method-related side effects were based on stories from their friends instead of medical information provided by a doctor. Additionally, providers were generally considered as more of an information source about specific methods, after the decision to seek a method had already been made. This idea should push programming to think more broadly on how to target the popular health sector that adolescent girls look to in their decision to seek a method.

This analysis also found that when deciding to seek a method, girls look to peers and partners for support. Parents’ influence was more mixed, as they were perceived of as a valuable resource, but also challenged by community norms. Each of these influencers plays a different role; harnessing these roles with targeted interventions can ensure that adolescents are getting accurate information and support. For example, we found that fears around side effects commonly emerged from hearing stories from peers and parents, while the weight of these effects were perpetuated by the social importance placed on fertility and marriage. Our findings are consistent with other programs that have harnessed peer and parental support for adolescent and youth contraceptive use. For example, the GREAT project in Uganda created spaces for community dialog to spark conversation within peer groups, using radio shows with characters to deliver the relevant positive messages [28]. These types of programs may lead to peer discussions focusing on facts and not just myths and this can be influential, since the role of peers on contraceptive decision-making is important [29]. That said, more research is needed in how to best utilize parental influence, as it is less studied than peer influence [30].

Our findings identified the important role that a partner can play in the contraceptive decision-making process, including being a potential asset in breaking down barriers such as cost. In developing the program, the NURHI team found that communication with husbands, among women of all ages, increased the likelihood of use [16]. The PRACHAR program found that young couples that went through the group training after marriage, had an increased use in contraception [31]. While these programs found a positive impact of engaging partners, it is important in the future, that the autonomy of decision-making is also considered. Despite the role of the partner being mentioned less often, the decision to seek care was described by FGD participants as almost completely placed in the hands of a partner when the partner was involved.

The vignette structure of the interview guide was chosen to help identify injunctive norms that serve as influencers and barriers in the community and how they influence the perceived descriptive norms. The vignette structure permits FGD participants to use the character to discuss their perspectives in a less personal way. However, given the scenarios provided, it did not elicit discussions about other potential barriers for adolescents. For example, physical barriers like proximity to a method source, the ability to purchase contraceptives in drug shops, or lack of youth friendly services were not discussed by the participants, despite prior studies that have demonstrated these to be barriers or facilitators to adolescent access in previous research [32–34]. To investigate these issues further, future research should explore the pathways to care with a particular emphasis not only on influences, but also including the physical process of accessing a method as well as what factors influence selection of a specific source. Another challenge with the vignette structure was its potential inflation of a girl’s ability to defy the established injunctive norm. With the vignette exhibiting a character who is defying these norms, the participants are primed to see girls in the community through this lens. This scenario may have promoted more examples of girls breaking through these norms than are actually happening. Based on national survey data, the average age of first contraceptive use is not until 24.9 years, suggesting that this type of priming may have occurred [17]. To prevent this type of potential priming in the future, creating an additional vignette that more closely mirrors population level demographics may allow for FGD participants to connect the characters to the people around them. Finally, due to the use of different moderators, the quality of data differed between regions. Though examples across regions are exhibited above, the perceptions of some groups with less rich information may be under-represented in our analysis.

## Conclusion

Our findings suggest that despite established community norms that create a lack of acceptance for contraceptive use by unmarried adolescents and youth and low overall use within the population, young women with a supportive social network can navigate barriers to access contraceptive methods. Programming that promotes open conversations between adolescents and their peers and partners can influence a girl’s contraceptive decision-making. This could happen through promotion of discussion through mass media, community outreach, or through school-based programming. What this research shows is that the creation of policies and programs that target the described popular health sector, in combination with activities at the community level, can ensure the Nigerian family planning efforts are targeting critical influences on adolescent girls’ care-seeking behaviors, and help facilitate improved health and well-being of young people for the long-term.

## Data Availability

The datasets generated and/or analyzed during the current study are not publicly available in order to protect the identities of the participants involved but are available from the second author on reasonable request.

## References

[1] Lloyd CB, Mensch BS (2008). Marriage and childbirth as factors in dropping out from school: an analysis of DHS data from sub-Saharan Africa. Popul Stud.

[2] Rosenberg M (2015). Relationship between school dropout and teen pregnancy among rural South African young women. Int J Epidemiol.

[3] Darroch JE, et al. Adding it up: costs and benefits of meeting contraceptive needs of adolescents. Guttmacher Institute. 2016. https://www.guttmacher.org/sites/default/files/report_pdf/adding-it-up-adolescents-report.pdf.

[4] Fikree FF (2017). Making good on a call to expand method choice for young people—turning rhetoric into reality for addressing Sustainable Development Goal Three. Reprod Health.

[5] Chandra-Mouli V (2013). Invest in adolescents and young people: it pays. Reprod Health.

[6] Global Consensus Statement for expanding contraceptive choice for adolescents and youth to include long-acting reversible contraception. https://www.familyplanning2020.org/sites/default/files/Global%20Consensus%20Statement%20-%20Expanding%20Contraceptive%20Choice.pdf. Accessed 31 Aug 2020.

[7] World Health Organization. WHO guidelines on preventing early pregnancy and poor reproductive outcomes among adolescents in developing countries. 2011. https://apps.who.int/iris/bitstream/handle/10665/44691/9789241502214_eng.pdf?sequence=1. 26180870

[8] MacQuarrie, Kerry LD. Unmet need for family planning among young women: levels and trends. DHS Comparative Reports No. 34. Rockville, Maryland, USA: ICF International. 2014.

[9] Kilanowski JF (2017). Breadth of the socio-ecological model. J Agromed.

[10] Harris S, Pierce M, Madsen EL, Gilles K. Youth Family Planning Scorecard. 2017. https://www.prb.org/Publications/Reports/2017/Global-Youth-Family-Planning-Index.aspx.

[11] Hussain R (2016). Unmet need for contraception in developing countries: examining women’s reasons for not using a method.

[12] Chandra-Mouli V, et al. Contraception for adolescents in low and middle income countries: needs, barriers, and access. BMC Reprod Health. 2014. https://download.springer.com/static/pdf/115/art%253A10.1186%252F1742-4755-11-1.pdf?. 10.1186/1742-4755-11-1 PMC3882494 24383405

[13] Calhoun LM (2013). Provider imposed restrictions to clients’ access to family planning in urban Uttar Pradesh, India: a mixed methods study. BMC Health Serv Res.

[14] Sinai I, et al. Programmatic implications of unmet need for contraception among men and young married women in northern Nigeria. Open Access J Contracept. 2018. 10.2147/OAJC.S172330 PMC6236097 30519126

[15] Oluwasanu MM (2019). Access to information on family planning and use of modern contraceptives among married Igbo Women in Southeast, Nigeria. Int Q Community Health Educ.

[16] Nigerian Urban Reproductive Health Initiative, theory application. 2013. https://www.nurhitoolkit.org/theory-application#.XGDDYs9KgWo.

[17] PMA 2020. Adolescents & youth adults health brief. PMA 2020/Nigeria, 2017:1–2.

[18] National Population Commission of Nigeria. Nigeria Demographic and Health Survey, 2013. 2014. 10.1111/j.1728-4465.2008.00154.x.

[19] Speizer IS, Lance P (2015). Fertility desires, family planning use and pregnancy experience: longitudinal examination of urban areas in three African countries. BMC Pregnancy Childbirth.

[20] Whitehouse B, Hollos M (2014). Definitions and the experience of fertility problems: infertile and sub-fertile women, childless mothers, and honorary mothers in two southern Nigerian communities. Med Anthropol Q.

[21] Bailey A. Let’s tell you a story: use of vignettes in focus group discussions on HIV/AIDS among migrant and mobile men in Goa, India. In: Doing cross-cultural research: ethical and methodological perspectives. 2008.

[22] Gourlay A (2014). Using vignettes in qualitative research to explore barriers and facilitating factors to the uptake of prevention of mother-to-child transmission services in rural Tanzania: a critical analysis. BMC Med Res Methodol.

[23] Cialdini RB (1990). A focus theory of normative conduct: a theoretical refinement and re-evaluation of the role of norms in human behaviors. J Pers Social Psychol.

[24] Kleinman A (1980). Patients and healers in the context of culture: an exploration of the borderland between anthropology, medicine, and psychiatry.

[25] Cartwright AF, Otai J, Maytan-Joneydi A (2019). Access to family planning for youth: perspectives of young family planning leaders from 40 countries. Gates Open Res.

[26] Nash K, O'Malley G, Geoffroy E, Schell E, Bvumbwe A, Denno DM (2019). "Our girls need to see a path to the future"—perspectives on sexual and reproductive health information among adolescent girls, guardians, and initiation counselors in Mulanje district, Malawi. Reprod Health.

[27] Mushy SE, Tarimo EAM, Fredrick Massae A, Horiuchi S (2020). Barriers to the uptake of modern family planning methods among female youth of Temeke District in Dar es Salaam, Tanzania: a qualitative study. Sex ReprodHealthc.

[28] Institute for Reproductive Health—Georgetown University. The GREAT PROJECT. 2015.

[29] Iyoke C (2014). Peer-driven contraceptive choices and preferences for contraceptive methods among students of tertiary educational institutions in Enugu, Nigeria. Patient Prefer Adherence.

[30] Guilamo-Ramos V (2016). Parent-adolescent communication about contraception and condom use. JAMAPediatr.

[31] Wilder J, Masilamani R, Daniel E. Promoting change in the reproductive behavior of youth—pathfinder International’s PRACHAR Project, Bihar, India.

[32] Shiferaw S (2017). Does proximity of women to facilities with better choice of contraceptives affect their contraceptive utilization in rural Ethiopia?. PLoS ONE.

[33] Benson A (2017). Longitudinal evaluation of the tupange urban family planning program in Kenya. Int Perspect Sex Reprod Health.

[34] Rosenberg NE (2018). Comparing youth-friendly health services to the standard of care through “Girl Power-Malawi”: a quasi-experimental cohort study. J Acquir Immune DeficSyndr.

